# A pipeline to extract drug-adverse event pairs from multiple data sources

**DOI:** 10.1186/1472-6947-14-13

**Published:** 2014-02-24

**Authors:** SriJyothsna Yeleswarapu, Aditya Rao, Thomas Joseph, Vangala Govindakrishnan Saipradeep, Rajgopal Srinivasan

**Affiliations:** 1 TCS Innovation Labs, Tata Consultancy Services Ltd, Deccan Park, 1, Software Units Layout, Madhapur, Hyderabad 500081, Andhra Pradesh, India

**Keywords:** Pharmacovigilance, NLP, Text mining, Social media, Adverse event, Biomedical literature, Unstructured text, BCPNN

## Abstract

**Background:**

Pharmacovigilance aims to uncover and understand harmful side-effects of drugs, termed adverse events (AEs). Although the current process of pharmacovigilance is very systematic, the increasing amount of information available in specialized health-related websites as well as the exponential growth in medical literature presents a unique opportunity to supplement traditional adverse event gathering mechanisms with new-age ones.

**Method:**

We present a semi-automated pipeline to extract associations between drugs and side effects from traditional structured adverse event databases, enhanced by potential drug-adverse event pairs mined from user-comments from health-related websites and MEDLINE abstracts. The pipeline was tested using a set of 12 drugs representative of two previous studies of adverse event extraction from health-related websites and MEDLINE abstracts.

**Results:**

Testing the pipeline shows that mining non-traditional sources helps substantiate the adverse event databases. The non-traditional sources not only contain the known AEs, but also suggest some unreported AEs for drugs which can then be analyzed further.

**Conclusion:**

A semi-automated pipeline to extract the AE pairs from adverse event databases as well as potential AE pairs from non-traditional sources such as text from MEDLINE abstracts and user-comments from health-related websites is presented.

## Background

With the large and growing set of medication drugs, it is very essential to assess the effects of medication on the patient population at large via information gathering and analysis. Since there are practical limits on the degree to which safety of drugs can be established prior to marketing approval, it is only through such mechanisms that can we understand the safety and harmful side effects of administered drugs. Typically, pre-marketing safety studies such as clinical trials are spread over a short duration and restricted to a small and mostly homogeneous study population. Furthermore, side effects of drugs are often revealed after the drug is out in the market being administered to a population, sometimes concomitantly with other drugs. Thus, it is critically important to constantly monitor the safety of drugs that have been launched in the market. To provide an objective basis for assessing the safety of marketed drugs, regulatory agencies have in place a post-marketing surveillance mechanism called Pharmacovigilance (PV) [1]. As per World Health Organization (WHO), PV is defined as *“the science and activities relating to the detection, assessment, understanding, and prevention of adverse effects or any other drug-related problems”*[2,3].

PV is required for systematically identifying causal associations between drugs and side-effects and taking corrective actions, both for new drugs being launched, as well as for drugs already in use. It is based on the collection of spontaneously reported Adverse Event (AE) reports. Report initiation by health professionals and consumers is generally voluntary. However, pharmaceutical companies are legally obliged to follow up on reports received, and to cascade these to various regulatory authorities [3,4]. The Adverse Event Reporting System database maintained by the US Food and Drug Administration (FDA), formerly called the Adverse Event Reporting System (AERS) and now referred to as the FDA Adverse Event Reporting System (FAERS) [5], collates all such reports and makes them available to the public at large [6]. Adverse events can be reported by pharmaceutical companies, health-care professionals as well as the general public. Such Spontaneous Reporting Systems (SRS) have certain limitations such as the potential AE reports being incomplete or inaccurate as a result of voluntary reporting; cases of biased reporting or under-reporting; or “Patient Reporter Event and Drug” (PRED) requirements for submission, geographic marketing and population varying for different drugs [1].

Mining of probable AE pairs in the FDA SRS has also been well studied [6]. Various studies have also looked at mining potential AEs from unstructured text sources such as Electronic Health Records (EHR), health-related websites and the MEDLINE database [7], serving as a complement to the SRS systems. Some studies have been done taking into account electronic health record databases to analyze drug safety such as EU-ADR [8,9], OMOP [10], Mini-Sentinel [11] as well as databases such as MEDLINE and Drugbank [12]. These studies show that combining the same types of data from multiple sources could help in better detection of potential AEs. Of course, sources such as EHR are not easily accessible due to privacy concerns. Another study was done involving the knowledge collected from publicly available drug-related information sources, UMLS [13], FAERS and SemMed [14], focusing on the ‘drug-indication’ association [15]. A key aspect when detecting potential AEs or extracting facts from unstructured text is to verify the results manually. Other studies have focused on using machine learning-based systems for the identification and extraction of potential AE pairs from MEDLINE case reports and generation relevant corpora [16,17]. van Mulligen et al. [18] describe the creation of a database which contains associations between drugs, disorders and targets mined from MEDLINE abstracts. These associations at the sentence-level in texts were further refined and corrected using human annotators.

The motivation for using unstructured text from health-related websites to extract AE associations are a consequence of the recent trend of people tending to blog about their personal experiences more frequently than reporting them to physicians. Health-related websites allow people to discuss their medical conditions with one another. A system of informal support in terms of forums facilitates online discussions among people administered the same/similar drug. Patients and the general public write about treatment they are undergoing, as well as respond to queries on treatment, side effects and related issues [9]. Such blogs also serve as indicators to the usage of drugs which might not be strictly in accordance with the recommended practices. Monitoring the conversations on these websites can alert pharmaceutical companies and regulatory bodies across the world to potential AE. A study was conducted by annotating 3600 comments from the health-related website DailyStrength [19,20]. This study showed that though user-comments pose a significant Natural Language Processing (NLP) challenge, they do contain useful information which could be prove beneficial on further exploration.

The studies of Wang et al. [1] and Leaman et al. [20] are significant for mining potential AEs from unstructured text sources. Wang et al. focused on demonstrating the feasibility of using narrative text in EHRs and association statistics for PV to detect novel AEs using NLP. They used the MedLEE (*Med*ical *L*anguage *E*xtraction and *E*ncoding) system for extracting and encoding information in clinical narratives such as the discharge summaries of inpatients. Their study was built on their previous work by adapting a combination of NLP and statistical methods to acquire potential AE associations. A chi-square test adjusted with volume was used on the co-occurring AE pairs to determine possible signals from them [1]. This work provided a possible method to establish safety profiles from unstructured patient data for a drug during its market life. However, it does not leverage the large amount of data available in health-related websites.

Leaman et al. [20] studied the validity of identifying associations between drugs and AEs reported by patients in the user comments of health-related websites. They implemented an automatic web crawler in their study that efficiently gathered user comments about specific drugs from the DailyStrength website. A dictionary compiled from four different sources viz COSTART vocabulary [21], SIDER side effects [22], the Canadian Drug Adverse Reaction Database MedEffect [23] and UMLS concept identifiers [13] - was used to extract the adverse drug reactions from these user comments. This work concluded that while mining user comments does pose significant challenges, these comments contain information that could prove to be useful in PV. However, the study does not include mining MEDLINE abstracts, as also using AE pairs from traditional SRS databases.

There is a need for a pipeline that can integrate data from traditional SRS databases such as the FAERS, user-comments from health-related websites as well as MEDLINE abstracts to detect potential AEs and provide biological context to these potential AEs. These potential AE pairs should then be compared with those listed in the label information of the drugs. Finally, statistical techniques will be used to determine the significant AE pairs. Our objective in this study is to develop a pipeline that can handle these requirements.

## Methods

The following steps were carried out:

1. Creating the complete pipeline.

2. Running the pipeline on the sources of data like the MEDLINE abstracts and the user comments from health-websites, respectively. As a pre-processing step, this involved obtaining the drug-AE pairs from each of the three sources. For MEDLINE abstracts and user-comments, these pairs are obtained using the Association Map module of PV-TPX. For the FAERS database, the drug pairs were obtained from the FDA datasets.

3. Running the BCPNN algorithm on the drug-AE pairs from individual sources.

4. Comparative analysis of the results from the BCPNN results in order to identify the potential adverse events for the drugs.

### TPX framework

We have previously developed TCS Pubmed eXplorer (TPX) [24], a web-based tool that supports concept-assisted search and navigation based on PubMed as the underlying search engine, to search the MEDLINE database. Although the focus of the TPX pipeline is better search of MEDLINE using PubMed, certain components of the TPX pipeline are generic and can be re-used in many biomedical tasks. We have taken relevant components of TPX for the semi-automated pipeline for AE event detection. In addition, we have developed new modules for tasks. TPX has 16 concept types, of these the drug, disease and symptom dictionaries were used in this study. Additionally, the annotation server was re-used with major modifications. A modified version of the TPX framework, hereon referred to as PV-TPX, was used in this study.

### Named-entity-recognition

One of the most significant tasks was the identification of drug and adverse event mentions in text from both MEDLINE as well as health-related websites using Named-Entity-Recognition (NER). The NER module of PV-TPX is based on that of TPX and uses dictionary-based NER techniques for identifying various biological entities in text. The PV-TPX NER module is part of the Annotation server that receives the unstructured textual content and performs a wide range of text-mining tasks. It was implemented in Java and used as a REST/SOAP based Web Service [25,26]. The following components of the NER module were used for processing the text:

•Part-Of-Speech (POS) Tagger: PV-TPX uses the Java implementation of the open source MedPost POS tagger [27] from NCBI, which is an HMM based POS tagger for parts-of-speech tagging in medical text.

•Stemmer and Tokenizer: PV-TPX uses the Porter stemmer algorithm for stemming [28]. An in-house implementation of tokenizer and sentence splitter is used for tokenization and sentence splitting respectively.

•Acronym Handler: PV-TPX also identifies local abbreviations by keeping track of such abbreviation definitions. The expansion of the abbreviations is usually specified in the article abstract, while the abbreviated form is used in the article title. Hence the abstract is tagged before the title and all the local abbreviations detected in the abstract were extended to the title tagging.

### Dictionary compilation

An inherent task for accomplishing NER was building the dictionaries to be used in the pipeline. While TPX uses dictionaries for various biological entities such as genes, proteins, diseases and drugs, none of them could be used as-is for this study. Instead, a *drug dictionary* and an *event dictionary* consisting of disease and symptom terms for identifying AEs were built as follows:

1. A *drug dictionary* is based on the TPX drug dictionary but enhanced with synonym/variants/brand names. The additional synonyms or variants for each of these that were compiled from sources such as MeSH [29]. Although not a formal input source, Wikipedia [30] data was used to cross-check some of the brand names as it proved to have significant brand names mentioned.

2. An *event dictionary* that consists of disease and symptom terms for identifying AEs. The event dictionary is primarily derived by merging *TPX disease dictionary* and *TPX symptom dictionary*[24]. Further, MedDRA [31] was used as an important source for enhancing the event dictionary since medication errors reported to FAERS are coded to terms in the MedDRA terminology. Hence, the MedDRA Preferred Term (PT) and Low Level Term (LLT) were also added to the event dictionary. To reconcile disparate mentions of named entities, normalization was done on the event dictionary as follows. For an exact match of the MedDRA term with the dictionary term, all the synonyms of that MedDRA PT were added as synonyms to the dictionary term. For MedDRA terms that do not have an exact match in the dictionary, the terms were added as separate entries in the dictionary. Only exact matches were handled here and no pattern matching was involved. For instance, MedDRA terms such as ‘abdominal discomfort’, ‘abnormal dreams’ and ‘acute psychosis’ that did not have an exact match in the dictionary were added to it as separate entries.

### Entity association module

TPX has a pairwise concept association module incorporated. The Concept association module reads the entity annotations for the entire MEDLINE and then computes pairwise associations between the biological entities. Thus, these associations are pre-computed and ranked according to their relevance to the whole of the tagged MEDLINE corpus. Additionally, the associations are scored based on co-occurrence within the abstracts. The scoring method is as follows: For an entity *e*, let *A*(*e*) = {*a*_1,_ …., *a*_*k*_} denote the set of abstracts *e* is mentioned. Let *t*_1_, …, *t*_*m*_ denote the set of all entities other than *e* mentioned in abstracts belonging to *A*(*e*). Let *A* denote the set of all abstracts. For an entity *t*_*i*_, let *N*(*t*_*i*_, *A*) denote the total number of occurrence of *t*_*i*_ in the abstracts in *A*. Similarly let *N*(*t*_*i*_, *A*(*e*)) denote the corresponding number of occurrence of *t*_*i*_ in the abstract collection *A*(*e*). The association score *P*(*t*_*i*_|*e*) denotes the probability that *t*_*i*_ is relevant given the entity *e*, is estimated using the standard tf-idf score as follows:

$$\begin{array}{l} P\left(t_{i}|e\right)=\frac{B\left(t_{i}|e\right)}{\sum_{j=1}^{m}B\left(t_{j}|e\right)}\\ \mathrm{where}\;B\left(t_{i}|e\right)=N\left(t_{i},A\left(e\right)\right)\log\left(\frac{N+1}{N\left(t_{i},\;A\right)}\right) \end{array}$$

where *N* is the sum total of the frequency of all the entities in the abstract collection *A*.

In PV-TPX, the pairwise associations between the identified entities were calculated using a PV-TPX specific pairwise entity association map. These pairwise associations are computed for the MEDLINE corpus as described above, where each abstract accounts for one document. However, each individual user comment and its responses from the health-related websites is considered as one document while computing the pairwise associations for this corpus. The resultant set for each corpus contains the drug-disease and drug-symptom pairs which were then processed to identify the potential AE pairs.

### BCPNN algorithm

The statistical BCPNN algorithm is used as the means for signal detection in the pipeline. It uses a neural network architecture to measure dependencies between entities in a dataset of AE pairs. BCPNN can be used to detect unexpected patterns in input data and to examine how such patterns vary over time [32]. It uses a disproportionality measure known as Information Component (IC). In BCPNN, node activations represent probability or confidence in the presence of input features, and synaptic weights are based on estimated correlations and the spread of activation corresponds to calculating posterior probabilities [33]. The variance values are relevant when the data is varying and not static.

The BCPNN algorithm has been implemented in Java for this pipeline, which takes as input, a matrix of the drug-AE associations and their frequency of occurrence in that particular source. The output from this is an IC variance value for each drug-AE pair, which is analyzed manually to obtain the potential drug-adverse event pairs. After the identification of drug and symptom or disease pairs from each source, this data arranged in a matrix form, which is the required input format for running the BCPNN algorithm. This implementation provides the ‘variance’ values for the IC for each of the AE pairs. The IC value in each source is based on the total number of documents in the set with drug X (C_x_); the total number of documents with AE entity Y (C_y_); the number of documents with the specific AE combination (C_xy_); and the total number of documents in that source. A variation in the data may cause the IC to either increase or decrease. The standard deviation for each IC provides a measure of the robustness of the value. Large values of C_x_, C_y_ and C_xy_ indicate smaller confidence intervals.

The IC is thus a measure of the strength of the dependency between a drug and an AE [33]. A positive IC value indicates that a particular AE combination is reported to the database more often than expected from the rest of the reports in the database. An IC value of zero indicates that there is no quantitative dependency between the AE combinations while a negative IC value indicates that the combination is reported to the database less frequently than statistically expected. The higher value of the IC, the more the combination stands out from the background. If the IC value increases over time and the value is positive, the positive quantitative association between the drug and the adverse e is likely to be high.

### Data acquisition

Data acquisition, which involved identifying the structured and unstructured sources of data for these drugs and collecting the data to be mined from them. Data acquisition from unstructured sources was done using the PV-TPX pipeline, which in turn involved processing the data from the different sources to identify entities and finding the associations amongst these entities. The drug and event dictionaries were used to identify entities.

### Structured-data acquisition

The FAERS database was used as the source of structured data. The FDA releases Adverse Event Reaction information on a quarterly basis. The drug, reaction, indication, outcomes, report sources, therapy and demographics files have been extracted from each quarter’s archive and loaded into a relational database. The drug-AE pairs have been obtained from the drug and reaction tables based on the Individual Safety Report (ISR) field. Duplicates were eliminated and unique drug-AE pairs were obtained for each ISR and case-id. Demographics information such as ISR, case-id, initial or follow-up code, age, gender, event date and reporting date were used for selecting unique records. The initial and follow-up cases were considered as two different instances of the association.

Before performing association mining on the data, normalization was done by comparing each of the drug and AE entities with the baseline dictionary and using the generic names or common synonyms for them. If the reaction or AE entity matched with the dictionary term, then the dictionary entry was used, otherwise the reaction entity was used *as-is* for further processing.

The FAERS files from the first quarter of 2008 to the first quarter of 2012 were obtained from the FDA website for this study. Therefore, the data was divided into three categories to observe the variance. The three categories are:

1. The complete set of data for each data source: FAERS data for all quarters from Q1 2008 to Q1 2012, all the blogs from the three health-related websites and all the abstracts from MEDLINE.

2. Data between January 01, 2008 and December 31, 2009: The FAERS drug-PT pairs with the FDA reported date within the given date range, the blogs from PatientsLikeMe and Mediguard with dates in the given range, the complete Dailystrength blogs and AE pairs from MEDLINE abstracts that have publication dates in the given range.

3. Data between January 01, 2010 and March 31, 2012: Similar to the above set.

### Unstructured-data acquisition

**Figure 1:**
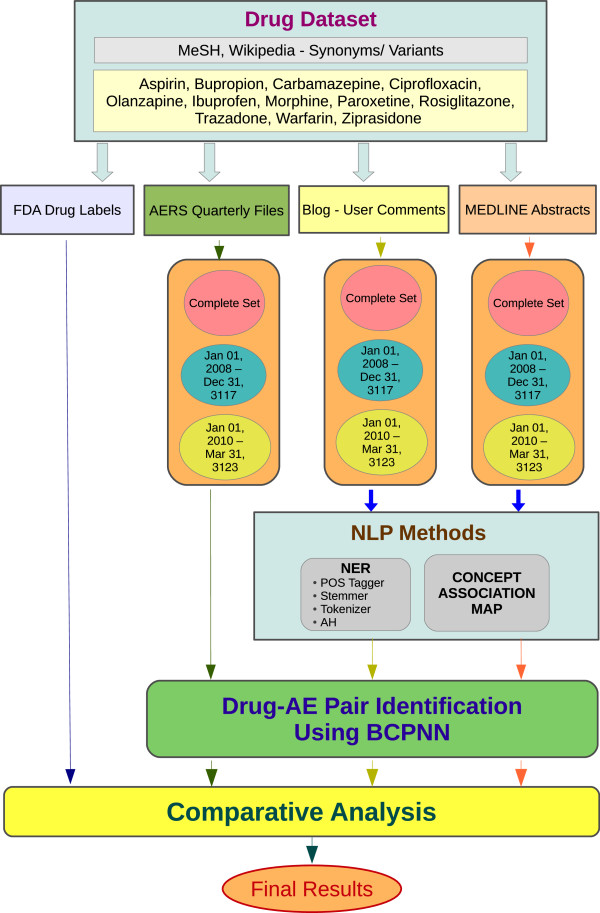
The pipeline depicting the sequential steps to identify the AE pairs from the various sources.

The user comments from health-related websites “PatientsLikeMe” [34], DailyStrength [19] and MediGuard [35] were used as sources of unstructured text. These blogs were crawled using Web-Harvest, an open-source web data extraction tool [36]. The user comments, reviews and replies were retrieved from these websites for the predefined set of drugs. These blogs also provide some user information such as the user-name, age, gender and demographics. However, since the study does not aim at categorizing the results based on these parameters, none of this additional information was used for processing. The other source of unstructured text is the MEDLINE database, which has over 23 million abstracts [7] Figure 1.

### Testing the pipeline

The pipeline takes about 36 hours for processing the entire MEDLINE corpus and nearly 20 minutes for the 13,500 user comments from the health-related websites. This includes the steps involving NER tasks such as POS tagging, stemming, tokenization, acronym handling and computing the entity association pairs.

A set of 12 drugs, which is the union of drugs that were used by Wang et al. [1] and Leaman et al. [20] in their study, was used for testing the pipeline (Table 1). The drugs include: Bupropion, Carbamazepine, Olanzapine, Ciprofloxacin, Trazodone, Ziprasidone, Aspirin, Ibuprofen, Warfarin, Paroxetine, Rosiglitazone and Morphine. Bupropion is an atypical anti-depressant and a smoking cessation agent [37]. Carbamazepine, Olanzapine, Trazadone, Ziprasidone and Paroxetine are different kinds of anti-psychotic or anti-depressant drugs. Ciprofloxacin is an anti-biotic; Aspirin, Ibuprofen and Morphine are different types of analgesic or anti-inflammatory drugs. Rosiglitazone is an anti-diabetic and Warfarin is an anticoagulant.

**Table 1:** Details of the drugs, as indicated by Wang et al. and Leaman et al., used in the current work

**No**	**Drug name**	**Drug type**	**Synonyms**	**Indications**	**Known AEs**
1	Bupropion	Atypical anti-depressant and smoking cessation agent	Wellbutrin, Quomen, Zyntabac, Bupropion hydrochloride, Zyban, Amfebutamone, Voxra, Budeprion, Aplenzin	Depression and smoking cessation aid	Dizziness, abnormal sensation, difficulty, drugged state, fatigue, constipation sleeplessness, seizure, tinnitus, pruritus feeling suicidal
2	Carbamazepine	Antipsychotic/ anti-depressant	Carbamazepine hydrochloride, Tegretol, Carbazepin, Carbamazepine sulfate (2:1), Carbamazepine dihydrate, Amizepine, Amizepin, Finlepsin, Neurotol, Epitol, Carbamazepine acetate, Carbamazepine phosphate, Biston, Calepsin, Carbatrol, Equetro, Sirtal, Stazepine, Telesmin, EPITAB XR, Teril, Timonil, Trimonil, Epimaz, Carbama, Carbamaze, Carzine, Mazetol, Tegrita, Tegrital, Karbapin, Hermolepsin, Degranol, Tegretal, Mannomustine, Mannitlost, Decranol, Mannitol mustard	Epilepsy, trigeminal neuralgia	Dizziness, somnolence or fatigue, unsteadiness, nausea, vomiting
3	Olanzapine	Antipsychotic/ anti-depressant	Zyprexa, Zydis, Relprevv, Zyprexa Relprevv	Schizophrenia, bipolar disorder	Weight gain, alteration in lipids, somnolence or fatigue, increased cholesterol, diabetes
4	Ciprofloxacin	Antibiotic	Ciprofloxacin hydrochloride, Ciprinol, Cipro, Baycip, Ciloxan, Ciflox, Cipro XR, Cipro XL, Ciproxin, Prociflor, Proquin, Proquin XR, Ciprex, Cetraxal, Axcin	Bacterial infection	Diarrhea, vomiting, abdominal pain, headache, restlessness
5	Trazadone	Antipsychotic/anti-depressant	Trazon, Thombran, Trazodone hydrochloride, Gen trazodone, Trazodon neuraxpharm, Molipaxin, Apo trazodone, Trittico, Deprax, Novo trazodone, Pms-trazodone, Nu-trazodone, Nu trazodone, Desyrel, Oleptro, Beneficat, Desirel, Trazorel, Trialodine, Mesyrel	Depression	Somnolence or fatigue, headache, dry mouth, dizziness, nausea
6	Ziprasidone	Antipsychotic/anti-depressant	Ziprasidone hydrochloride, ziprasidone hydrochloride, monohydrate, Geodon, Ziprazidone, Zeldox	Schizophrenia	Somnolence or fatigue, dyskinesia, nausea, constipation, dizziness
7	Aspirin	Analgesic/anti-inflammatory	Zorprin, Magnecyl, Acetylsalicylic acid, Polopirin, Solupsan, Endosprin, Polopiryna, Acetysal, Easprin, Ecotrin, Aloxiprimum, Colfarit, Dispril, Solprin, Micristin, Acylpyrin, Empirin, Bufferin, Fasprin, Genacote, Halfprin	Pain, fever, reduce blood clotting	Nausea, vomiting, ulcers, bleeding, stomach pain or upset
8	Ibuprofen	Analgesic/anti-inflammatory	Salprofen, Trauma-dolgit gel, Trauma dolgit gel, Rufen, Nuprin, Brufen, Motrin, Ibumetin, Nurofen, Advil	Pain of rheumatoid arthritis, osteoarthritis, menstrual cramps, or mild to moderate pain	Headache, achalasia, nausea, constipation
9	Warfarin	Anticoagulant	Coumadine, Tedicumar, Warfant, Coumadin, Gen-warfarin, Aldocumar, Marevan, Apo-warfarin, Jantoven, Lawarin, Waran	-	-
10	Paroxetine	Antipsychotic/ anti-depressant	Seroxat, Paroxetine maleate, Paxil, Aropax, Paroxetine acetate, Paroxetine hydrochloride, Paxil cr, Pexeva, Sereupin	Mental depression, obsessive-compulsive disorder, panic disorder, generalized anxiety disorder, social anxiety disorder	Pain chest, drowsiness, orthostasis, dyspnea, agitation, dizziness, feeling suicidal
11	Rosiglitazone	Antidiabetic	Avandia, Rosiglitazone maleate,	Diabetes	Headache, chest pain, left atrial hypertrophy, shortness of breath
12	Morphine	Analgesic/anti-inflammatory	Morphia, Oramorph SR, Duramorph, Morphine chloride, Morphine sulfate, Ms contin, Mir, Morin, Nepenthe, Mirs, Micro-rna, Avinza, Kadian, Morphine ir, Msir, Roxanol, Infumorph, Kapabloc, Kapanol, Loceptin, Longphine, Malfin, Maxidon, Meconium, Meslon, Micro-morphine, Mogetic, Morapid, Moraxen, Morcap, Moretal, Morfenil, Morficontin, Morfin, Morfin meda, Morfina, Morph, Morphanton, Morphex, Morphgesic, Morphin, Morphini, Morphinum, Morphiphar, Morphitec, Morphium, Morstel, Mos, Moscontin, Morstel, Mortificontin, Ms direct, Ms long, Ms mono, Mst continus, Mst unicontinus, Mundidol, Mxl, Neocalmans, Noceptin, Oblioser, Oglos, Oms concentrate Onkomorphin, Opitard, Opsalvina, Oramorph, Ordine, Relimal, Relipain, Repriadol, Rescudose, Sevredol, Skenan, Slovalgin, Srm-rhotard, Statex, Stellaphine, Stellorphinad, Stellorphine, Substitol, Vendal, Zomorph	-	-

The label information for each of these 12 drugs was obtained from the FDA website. These sections containing the prescribing or label information vary from drug-to-drug. Therefore depending on the information available in the files obtained from the FDA website, the sections used were the “Adverse Reactions”, “Warnings”, “Boxed Warning”, “Precautions” and “Use in Specific Populations”. A comparative analysis of the label information for each of the drugs was done with the AE pairs obtained.

The BCPNN algorithm was applied to the AE associations from the three sources - FAERS, health-related websites and MEDLINE abstracts. The AE pairs with positive variance values across these categories were considered as potential signals for further analysis. PERL scripts were used to create a tabular view of results for comparison and analysis. Further, the results were grouped manually for reporting.

## Results

Table 2 shows the results of the pipeline for Bupropion, in comparison with the results indicated by Wang et al. A comparative analysis of the results is reported for Bupropion as a representative out of the 12 drugs used for testing the pipeline. Wang et al. depict their qualitative evaluation of the results under four classes of associations into which the experts categorize the results obtained from their methods. The results for the other drugs is available as supplementary file (Additional file 1).

•Reference standard, which is constructed by the physician and Known AEs

•Indication Associations

•Remote Indication Association

•Unknown Associations

**Table 2:** A comparison of the results from the pipeline with those from the study of Wang et. al

**BUPROPION**				
**(Treatment indications: depression and smoking cessation aid)**				
	**Reference standard**	**Known AEs**	**Indication associations**	**Remote indication associations**
**Wang et. al**	Constipation, dizziness, drowsiness, dry mouth, headache, pruritus, increased sweating, loss of appetite, nausea, vomiting, nervousness, restlessness, taste changes, trouble sleeping, weight changes, seizure	Dizziness, abnormal sensation, difficulty, fatigue, constipation, sleeplessness, seizure, tinnitus, pruritus, feeling suicidal, drugged state	Suicidal, visual hallucinations, moody, emotional, tremor, nightmare	Motor retardation, fall, jumpy, stiffness, early satiety, extrapyramidal sign, energy increased, malingerer, rale, urge incontinence, bulimia, yellow sputum, emaciation
**Results from the pipeline**				
**Blogs**	Constipation, dizziness, drowsiness, dry mouth, headache, pruritus, eating disorders, anorexia nervosa, nausea, vomiting, shaking, hysteria, taste disorders, eating disorders, sleep deprivation, sleep initiation and maintenance disorders, sleep disorders, sleep apnea syndrome, weight loss, weight gain, overweight, weight, seizures, tinnitus, depression, major depression, stress disorders post-traumatic, anxiety	Dizziness, fatigue, constipation, sleep deprivation, sleep initiation and maintenance disorders, sleep disorders, sleep apnea syndrome, seizures, tinnitus, pruritus, depression, major depression, stress disorders post-traumatic, anxiety	Depression, major depression, stress disorders post-traumatic, anxiety, hallucinations, mood disorders, tremor, bad dreams, vivid dreams	Psychomotor agitation, eating disorders, weight loss
**MEDLINE Abstracts**	Constipation, dizziness, sleepiness, dry mouth, headache, pruritus, aquagenic pruritus, brachioradial pruritus, generalized pruritus, sweating, eating disorders, increased appetite, nausea, nausea and vomiting, vomiting, restless legs syndrome, taste disorders, taste disturbance, sleep deprivation, sleep initiation and maintenance disorders, sleep disorders, sleep apnea syndromes, sleep disturbances, sleep maintenance insomnia, sleep arousal disorders, loss of weight, weight increase, overweight, weight, body weight changes, seizures, atonic seizures, complex partial seizures, psychomotor seizures, alcohol withdrawal seizures, partial seizures, neonatal seizures, seizures febrile, tinnitus, suicidal behavior, suicidal ideation	Dizziness, sensation disorders, voiding difficulty, fatigue, mental fatigue, fatigue syndrome chronic, constipation, sleep deprivation, sleep initiation and maintenance disorders, sleep disorders, sleep apnea syndromes, sleep disturbances, sleep maintenance insomnia, sleep arousal disorders, seizures, atonic seizures, complex partial seizures, psychomotor seizures, alcohol withdrawal seizures, partial seizures, neonatal seizures, seizures febrile, tinnitus, pruritus, suicidal behavior, suicidal ideation, drug toxicity, drug-specific antibodies, drug screen, abnormalities drug-induced, drug diversion, drug intolerance, drug-induced headache, drug-induced lupus erythematosus, drug seeking behavior, drug overdose, akathisia drug-induced, dyskinesia drug-induced, multiple-drug resistance, fixed drug eruption, extensively drug-resistant tuberculosis, drug eruptions, drug-induced liver injury, drug resistance, drug hypersensitivity	Suicidal behavior, suicidal ideation, hallucinations, mood disorders, mood swings, depressed mood, emotional liability, tremor, holmes tremor, essential tremor, nightmares	Psychomotor agitation, psychomotor retardation, psychomotor disorders, peripheral sensorimotor neuropathy, psychomotor seizures, epilepsy partial motor, motor fluctuations, rhinitis vasomotor, oculomotor nerve diseases, fall, fear of falling, extrapyramidal symptoms, moist rales, urinary urgency, urination disorders, urinary bladder overactive, bulimia, bulimia nervosa, binge-eating disorder, eating disorders, sputum, weight loss, loss of weight
**AERS**	Constipation, dizziness, dizziness postural, dry mouth, lip dry, headache, pruritus, pruritus generalised, pruritus genital, instillation site pruritus, application site pruritus, ear pruritus, vulvovaginal pruritus, anal pruritus, eye pruritus, infusion site pruritus, injection site pruritus, night sweats, cold sweat, decreased appetite, appetite disorder, increased appetite, nausea, vomiting, vomiting neonatal, nervousness, restlessness, restless legs syndrome, eating disorder, product taste abnormal, sleep disorder, sleep apnoea syndrome, rapid eye movements sleep abnormal, sleep terror, irregular sleep phase, sleep phase rhythm disturbance, abnormal sleep-related event, poor quality sleep, weight decreased, weight increased, overweight, weight abnormal, weight loss poor, atonic seizures, complex partial seizures, partial seizures, tinnitus, depression suicidal, suicidal ideation, suicide attempt, suicidal behaviour	Dizziness, dizziness postural, abnormal sensation in eye, burning sensation, fatigue, constipation, sleep disorder, sleep apnoea syndrome, rapid eye movements sleep abnormal, sleep terror, irregular sleep phase, sleep phase rhythm disturbance, abnormal sleep-related event, poor quality sleep, atonic seizures, complex partial seizures, partial seizures, tinnitus, pruritus, pruritus generalised, pruritus genital, instillation site pruritus, application site pruritus, ear pruritus, vulvovaginal pruritus, anal pruritus, eye pruritus, infusion site pruritus, injection site pruritus, suicidal behaviour, depression suicidal, suicide attempt, suicidal ideation, drug intolerance, drug hypersensitivity, drug interaction, intentional drug misuse, drug withdrawal syndrome	Suicidal behaviour, depression suicidal, suicide attempt, suicidal ideation, completed suicide, hallucination visual, hallucination, hallucination olfactory, hallucination auditory, hallucinations mixed, hypnagogic hallucination, mood swings, depressed mood, elevated mood, mood altered, euphoric mood, emotional disorder, emotional distress, tremor, essential tremor, intention tremor, parkinsonian rest tremor, nightmare, abnormal dreams	Motor dysfunction, psychomotor retardation, psychomotor hyperactivity, fall, fear of falling, musculoskeletal stiffness, joint stiffness, early satiety, extrapyramidal disorder, energy increased, rales, incontinence, defaecation urgency, urinary incontinence, hypotonic urinary bladder, faecal incontinence, bulimia nervosa, binge eating, eating disorder, sputum discoloured, weight decreased, weight abnormal

We have used these categories of results for comparative purposes. Bupropion, however, according to the Wang et al. study does not have any *Unknown Associations*.

Table 3 shows the result of the comparative analysis of the pipeline results with the label information for Bupropion. Each row of the table shows different AEs grouped together, separated by a semicolon (;). The known AEs from label information for Bupropion, such as “nausea”, “dizziness” and “suicidal behavior” were identified. AEs such as “binge eating disorder”, which might lead to “diabetes mellitus, type 2”, were identified and rank high. Also, unique AEs were reported for Bupropion, such as “airway obstruction” and “breathlessness” in blogs, which might result from known reactions such as “angioedema”.

**Table 3:** Burproion: comparative analysis of the label information from FDA and the results of BCPNN on blogs, AERS and MEDLINE data

**BUPROPION**			
**Label**	**Blogs**	**AERS**	**MEDLINE**
Angioedema	Airway obstruction; hoarseness	Angioedema;	Angioedema
Myalgia	Fibromyalgia	Fibromyalgia; myalgia	–
Muscle rigidity; muscle weakness; musculoskeletal chest pain	Muscle cramps; myasthenia gravis	Muscle atrophy; muscle spasm; muscle strain; muscle twitching; musculoskeletal discomfort; musculoskeletal stiffness	–
Polyuria; dysuria; glucosuria	Proteinuria; hematuria	Nocturia; pollakiuria	–
Liver damage	Liver failure	–	–
Dry mouth; dry skin; dry eye;	Dehydration; dry mouth; dry eye syndrome;	Dry mouth; dry skin	Dry mouth; dry eye
Constipation; and headache	-	-	-
Chest pain	Breast neoplasms; neoplasms;	Breast cancer female; breast pain; drug exposure via breast milk; neoplasm; non-cardiac chest pain	Lung neoplasms
Neck pain	Back pain; intervetebral disc displacement; chronic pain; Neck pain	Back pain; neck pain; inter-vertebral disc injury; inter-vertebral disc protrusion	–
Dream abnormality	Bad dreams	Abnormal dreams	–
–	Constriction, pathologic	–	–
–	Contusions	–	–
–	Hypothyroidism; hashimoto disease	Hypothyroidism	–
–	Kidney diseases	–	–
Binge eating	Eating disorders; diabetes mellitus, type 2	Diabetes mellitus inadequate control; type 2 diabetes mellitus; type 1 diabetes mellitus	Binge-eating disorder, bulimia
Urinary frequency, urinary incontinence, urinary urgency, urinary retention, urine abnormality	–	Urine abnormality, metanephrine urine increased	–

One of the common trends noticed from these top scoring events for the drugs used in this study as listed in Table 4 is that in FAERS only the side effects or adverse events were high scoring, while in the other two sources some of the drug indications came up with a high score. This is possible because in these unstructured sources, the patients could have mentioned the reason for which the drug was administered or the early signs and symptoms they noticed for which the drug was prescribed.

**Table 4:** Top scoring adverse events for each of the drugs used in this study

	**Drugname**	**AERS**	**BCPNN score**	**Blogs**	**BCPNN score**	**Biomedical literature**	**BCPNN score**
1	Aspirin	FLUSHING COLITIS	3.0453446021	HEMORRHAGE	2.2974781367	ANTIPLATELET THERAPY	6.4498501796
		COLLAGENOUS	2.967777029	ASTHMA	1.7849414476	ASTHMA, ASPIRIN-INDUCED	6.2465967694
		GASTROINTESTINAL ULCER	2.8231226105	ULCER	1.6889750542	PLATELET AGGREGATION	4.9990559736
2	Bupropion	PSORIASIS	3.0858333183	SMOKING	2.8487222833	TOBACCO USE DISORDER	6.7616174378
		TINNITUS	3.0003865794	INTERACTION	2.0278154524	SUBSTANCE WITHDRAWAL SYNDROME	5.6160138431
		CRYING	2.9716117286	WEIGHT LOSS	1.9125982688	DEPRESSIVE DISORDER, MAJOR	5.0765060025
3	Carbamazepine	ANTICONVULSANT DRUG LEVEL INCREASED	3.8394557589	SEIZURES	2.1831882505	TRIGEMINAL NEURALGIA	5.7366534252
		DRUG RASH WITH EOSINOPHILIA AND SYSTEMIC SYMPTOMS	3.5911285749	EXANTHEMA	1.8627395675	EPILEPSIES, PARTIAL	5.5948458460
		EPILEPSY	3.0298491782	LYMPHOMA	1.4381580775	STEVENS-JOHNSON SYNDROME	5.3815350109
4	Ciprofloxacin	TENDON RUPTURE	4.1146259253	UTI	2.8856797854	MBC	4.6062736746
		TENDONITIS	4.0060829925	DIVERTICULITIS	2.2799989819	CROSS RESISTANCE	4.4062217226
		TENDON PAIN	3.6892637795	ACNE VULGARIS	1.7510888515	DYSENTERY, BACILLARY	4.3850295544
5	Ibuprofen	KOUNIS SYNDROME	2.9815043448	PAIN	2.3836377566	DUCTUS ARTERIOSUS, PATENT	4.8635855143
		TOXIC EPIDERMAL NECROLYSIS	2.8890346654	OSTEOARTHRITIS	1.9773610941	ACUTE PAIN	4.4671583670
		OSTEOARTHRITIS	2.8760959875	STOMACH ULCER	1.6554329992	OSTEOARTHRITIS	3.5868790708
6	Morphine	ACUTE CHEST SYNDROME	3.9896519753	HASHIMOTO DISEASE	0.9969507103	POSTOPERATIVE ANALGESIA	5.9041759822
		ACCIDENTAL DEATH	3.5380803319	BREATHLESSNESS	0.9969507103	OPIATES	5.8450420720
		CARDIO-RESPIRATORY ARREST	3.2009993755	VIOLENT	0.995428479	OPIOID-RELATED DISORDERS	5.4082024508
7	Olanzapine	HOSPITALISATION	3.6824877733	DIABETES MELLITUS	1.96163018	SCHIZOPHRENIA	5.7849079521
		LEUKOPENIA	3.2803137933	OVERWEIGHT	1.8868082131	BIPOLAR DISORDER	5.7118297960
		METABOLIC SYNDROME	3.1617168832	FEELING COLD	1.4527063824	PSYCHOMOTOR AGITATION	5.6353982109
8	Paroxetine	CONGENITAL ANOMALY	3.1300167523	ANXIETY DISORDERS	1.5594343083	HAM	5.6353454077
		ATRIAL SEPTAL DEFECT	3.1224780702	PHOBIC DISORDERS	1.5388697964	DEPRESSIVE DISORDER, MAJOR	5.5424233484
		CARDIAC MURMUR	2.9842039722	HOT FLASHES	1.4317963274	PANIC DISORDER	5.3198511794
9	Rosiglitazone	HEART INJURY	1.5296202559	HEART DISEASES	1.9460752101	DIABETES MELLITUS, TYPE 2	5.5082168188
		CARDIOVASCULAR DISORDER	1.5209213985	DIABETES MELLITUS	1.6536311321	INSULIN RESISTANCE	5.4173083948
		MYOCARDIAL ISCHAEMIA	1.5186640529	CORONARY ARTERY DISEASE	1.564503778	POLYCYSTIC OVARY SYNDROME	4.1594095171
10	Trazodone	CARDIAC ARREST	2.751062765	BACK PAIN	2.0113279316	SLEEP INITIATION AND MAINTENANCE DISORDERS	5.5423900660
		COMPLETED SUICIDE	2.4845999228	SLEEP INITIATION AND MAINTENANCE DISORDERS	1.4252916669	PRIAPISM	5.2601858298
		SUICIDAL IDEATION	2.3695413431	CONDYLOMATA ACUMINATA	1.3983875588	OVERDOSE	4.5116173724
11	Warfarin	INTERNATIONAL NORMALISED RATIO FLUCTUATION (INCREASED/ DECREASED)	3.1642485602	INTERNATIONAL NORMALIZED RATIO	4.2516503259	VITAMIN K	6.1945919883
		CALCIPHYLAXIS	2.8692951662	HEMORRHAGE	2.8983756918	BCR (BLOOD CLOTTING RESPONSE)	5.8376604098
		BLUE TOE SYNDROME	2.8692951662	STROKE	2.5276002364	THROMBOEMBOLISM	5.7963964427
12	Ziprasidone	TARDIVE DYSKINESIA	4.2677078217	VOMITING	1.9460228686	SCHIZOPHRENIA	5.7401521972
		DYSTONIA	4.0558212672	TREMOR	1.831079029	QT INTERVAL	5.6217692996
		EXTRAPYRAMIDAL DISORDER	3.7617945087	PSYCHOTIC DISORDERS	1.6030569698	BIPOLAR DISORDER	5.6013438249

The scores here are the variance values obtained from running the BCPNN algorithm on the drug-AE pairs.

Table 5 contains the top scoring adverse events across the three sources for anti-depressant/anti-psychotic class of drugs used in this study, which are prescribed for neurological problems. There are a few noticeable results. For example, side effects such as *cardiac disorders*, *sleep disorders* and *priapism* come up as high scoring ones for an anti-depressant drug such as Trazadone. *Heart diseases* also show up as high-scoring adverse events for an anti-diabetic drug such as Rosiglitazone.

**Table 5:** Top scoring adverse events for a class of drugs in this study, which are anti-depressant/ antipsychotic drugs prescribed for neurological problems, across the three different sources

**AERS**			**BLOGS**			**BIOMEDICAL LITERATURE**		
**DRUG**	**PT**	**BCPNN Variance**	**DRUG**	**EVENT**	**BCPNN Variance**	**DRUG**	**EVENT**	**BCPNN Variance**
ZIPRASIDONE	TARDIVE DYSKINESIA	4.2677078217	BUPROPION	SMOKING	2.8487222833	BUPROPION	TOBACCO USE DISORDER	6.761617438
ZIPRASIDONE	DYSTONIA	4.0558212672	CARBAMAZEPINE	SEIZURES	2.1831882505	OLANZAPINE	SCHIZOPHRENIA	5.784907952
CARBAMAZEPINE	ANTICONVULSANT LEVEL DRUG INCREASED	3.8394557589	BUPROPION	INTERACTION	2.0278154524	ZIPRASIDONE	SCHIZOPHRENIA	5.740152197
ZIPRASIDONE	EXTRAPYRAMIDAL DISORDER	3.7617945087	TRAZODONE	BACK PAIN	2.0113279316	CARBAMAZEPINE	TRIGEMINAL NEURALGIA	5.736653425
ZIPRASIDONE	AKATHISIA	3.6922456683	OLANZAPINE	DIABETES MELLITUS	1.96163018	OLANZAPINE	BIPOLAR DISORDER	5.711829796
OLANZAPINE	HOSPITALISATION	3.6824877733	ZIPRASIDONE	VOMITING	1.9460228686	OLANZAPINE	PSYCHOMOTOR AGITATION	5.635398211
CARBAMAZEPINE	DRUG RASH WITH EOSINOPHILIA AND SYSTEMIC SYMPTOMS	3.5911285749	BUPROPION	WEIGHT LOSS	1.9125982688	PAROXETINE	HAM	5.635345408
ZIPRASIDONE	BRUXISM	3.3493728888	OLANZAPINE	OVERWEIGHT	1.8868082131	ZIPRASIDONE	QT INTERVAL	5.621769300
OLANZAPINE	LEUKOPENIA	3.2803137933	CARBAMAZEPINE	EXANTHEMA	1.8627395675	BUPROPION	SUBSTANCE WITHDRAWAL SYNDROME	5.616013843
CARBAMAZEPINE	ANTICONVULSANT DRUG LEVEL ABOVE THERAPEUTIC	3.2657628102	ZIPRASIDONE	TREMOR	1.831079029	ZIPRASIDONE	BIPOLAR DISORDER	5.601343825

The scores here are the variance values obtained from running the BCPNN algorithm on the drug-AE pairs.

The results also show the association pairs Warfarin-INR, Warfarin-BCR and Warfarin-Vitamin K among the top scoring ones. Warfarin is an anti-coagulant commonly used to prevent *thrombosis* and *thromboembolism*, the formation of blood clots in the blood vessels and their migration elsewhere in the body, respectively. International Normalized Ratio (INR) is a measure of a pathway of coagulation while BCR is the test for blood clotting response. And, it is known that vitamin K is required for blood coagulation. Hence, the addition of MedDRA terms to the entity dictionary has contributed to Vitamin K showing up as a top scoring result, even though it is not really a disease or symptom term or even an adverse event by itself.

The supplementary material contains the comparative analysis of the pipeline results with the label information for some of the drugs, along with the top scoring results for the remaining drugs.

## Discussion

A semi-automated PV pipeline was built in this study, which includes an in-house application TPX. This pipeline serves as a framework for normalization of input from multiple sources and a semi-automated extraction of potential AE pairs from them. The pipeline was tested with a set of 12 drugs drawn from Wang et al. [1] and Leaman et al. [20]. An analysis of the top scoring results for each of these drugs shows that the three sources individually contribute to the identification of drug-adverse event pairs and that no single source caters completely to the potential drug-adverse event pairs all by itself. However, two patterns emerge from the comparative analysis of the BCPNN results of blogs with the label and FAERS results:

1. Some of the AEs reported in blogs were more specific when compared to the label information.

2. Some unique AEs were found in the health-related websites

The data sources used in this study are the validated set of AE pairs from FAERS, medical literature from MEDLINE and blog content health-related websites PatientsLikeMe, Mediguard and DailyStrength. These selected sources are only examples for each type of data and by no means implies that these are the authoratative sources for such data. There are other medical literature sources (PMC) or social media (other health-related websites) that could be of equal, if not more, value.

The blogs in health-related websites are not grammatically correct by nature. The language used can be very complex with varying writing styles. The format, structure, and style continuously evolve over time. Moreover, these blogs may not completely abide by the guidelines for AE reporting such as fulfilling the basic PRED criteria; which may not be entirely reliable. Also, they may not be validated by a trained investigator, the seriousness of the AE may not be explicitly specified, there might occur a considerable time-delay before being mentioned in the blog, they could be reported by a third-party or may have occurred concomitantly. However, the main topics of discussion in blogs of the health-related websites are medications, physiology and disorders. It has been observed that patients are concerned by medication, while physicians rather focus on illnesses [38].

Some components of the pipeline involve manual tasks. The pre-processing stage that involves data preparation contains a few manual steps. The user comments from the health-related websites were obtained using the Web-Harvest tool. This tool requires the scripts for data extraction to be written in xml. The user comments, thus obtained for each drug, were further broken down into individual records using a Perl script. PV-TPX is run on these individual records, each considered as a document. The post-processing stage, after the identification of drug and symptom or disease pairs, also involves some manual tasks such as preparing this data in a matrix form, which is the required input format for running the BCPNN algorithm. Also, the results obtained from the BCPNN algorithm were sorted based on their IC variance values and were then manually analyzed to identify the potential drug-adverse event pairs, for each drug across each of the sources.

Mining user comments from health-related websites helps avoid the time-consuming process of getting formal ethical approval for involving patients in direct primary research [39]. However, there is the issue of ethics as a concern while using data from such websites. In fact, a number of ethical considerations concerning the reporting of data obtained from the Internet have already been discussed and reported at great length by others [40-42]. There is however a growing consensus among researchers that if Internet data is freely and publicly accessible, then it can be used for considered research without prior approval. Based on this, data taken from the Internet have in fact been widely used already [39]. Most often users post to health-related websites with their user-names or other handles without disclosing any personal information. Since such personal information of the reporting person such as name, age and other demographics are not used or reported as part of the results of the study, the ethical issues such as privacy should not pose a significant concern.

Spam and malicious posts could affect to the content of such health-related websites and hence bias the results of any such analysis. However, unlike most regular blogs and bulletin boards, these health-related websites have moderators looking at content posted by users and removing such content, when encountered. For example, DailyStrength has a policy against both spammers and trolls. It defines spammers and trolls as follows: a spammer is someone who posts prolifically on dailystrength and on the comment systems in order to promote links or products, often of a commercial interest while a troll is someone who posts with the intent to rile up communities, provokes others into arguments or attacks, steers discussions off-topic and prevents them from being helpful, or disrupts and pollutes a support group with negativity.

There has been an increase in the number of commercial websites developed aiming to aggregate user comments from various health-related websites to obtain potential AE pairs. Treato is an example of one such website, which automatically collects the large amount of patient-written health experiences from health-related websites and uses advanced NLP to extract relevant information and create a comprehensive picture of what people say about their medications and conditions [43]. The website displays all the AE pairs as part of the results. An important aspect of our pipeline, when compared to Treato, is the grouping of AEs based on different criteria, such as synonyms or variants of other AEs. In the absence of an ontology for normalization, this step is critical for the identification and comparative analysis of AEs in the final results. Lack of such grouping results in presenting an incorrect picture. Treato, for example, displays the results for individual AEs separately with no grouping For example, “drowsy” and “sleepy” are reported as two separate AEs for Bupropion in Treato, thus displaying a larger number of AEs, even though they are inter-related.

The statistical algorithm BCPNN, which was applied in this work, generates association rules based on frequency. Many such quantitative methods have been used on SRS databases to detect and predict potential AE pairs. For example, the FDA uses Multi-item Gamma Poisson Shrinker (MGPS) to detect potential AE signals in its MedWatch program [44]. The UK Medicines Control Agency adopts Proportional Reporting Ratio (PRR) and Chi-square statistic to identify AE signals [45]. The Netherlands Pharmacovigilance Centre Lareb uses the Reporting Odds Ratio [46] and the Uppsala Monitoring Center employs Bayesian Confidence Propagation Neural Network (BCPNN) as its signal detection method on the WHO database [47]. While all these different methods have the ability to detect potential AE pairs, BCPNN is seen to have the best performance when compared to PRR and MGPS [48]. Hence, we decided to implement and incorporate BCPNN in our pipeline.

Of course, there are some drawbacks of using BCPNN or some statistical algorithms in general. The IC value in BCPNN does not give any information about the causality of an AE combination. The positive quantitative association between the drug and the AE is likely to be high, although clinical assessment remains essential. Typically, rare associations are not frequently reported. However, BCPNN works sufficiently well as the data augmentation from blogs and MEDLINE abstracts add up for these associations, which were traditionally viewed only from FAERS as rare ones. Hence, even to detect infrequent signals from multiple sources, we propose that our pipeline can be used.

The pipeline is not a fully-automated one. Some components of the pipeline involve manual tasks. The pre-processing stage that involves data preparation contains a few manual steps. The user comments from the health-related websites were obtained using the Web-Harvest tool. This tool requires the scripts for data extraction to be written in xml. The user comments, thus obtained for each drug, were further broken down into individual records using a Perl script. PV-TPX is run on these individual records, each considered as a document. The post-processing stage, after the identification of drug and symptom or disease pairs, also involves some manual tasks such as preparing this data in a matrix form, which is the required input format for running the BCPNN algorithm. Also, the results obtained from the BCPNN algorithm were sorted based on their IC variance values and were then manually analyzed to identify the potential drug-adverse event pairs, for each drug across each of the sources.

## Conclusion

We have built a semi-automated pipeline to extract the AE pairs from adverse event databases, enhanced by potential drug-adverse event pairs mined from non-traditional sources such as text from MEDLINE abstracts and user-comments from health-related websites. Testing the pipeline shows that although these non-traditional sources by themselves cannot be alternatives for AE detection, mining such sources helps substantiate the adverse event databases. They not only contain the known AEs, but also suggest unknown and unreported AEs for drugs, which can be analyzed further. While pharmaceutical companies may not want to incorporate these kinds of tools in their PV programs, the pipeline could prove to be useful for better PV by regulatory agencies, albeit with greater validations in place.

### Future work

The amount of automation in the pipeline can be increased, thus extending the semi-automated pipeline used for the current study. However, manual intervention will always be required. A weighting scheme for assigning more weight to associations from FAERS over associations from unstructured text can be designed and incorporated into the system before associations are reported. Another challenge is to successfully differentiate between indications, symptoms of these indications, and known contraindications. Also, a hierarchical representation or an ontology of the AEs can be built, in order to group them for further analysis.

## Competing interests

All the authors are salaried employees of Tata Consultancy Services Ltd. (TCSL), where this work was done. TCSL finances the article-processing charge for this manuscript. The authors have no other competing interests.

## Authors’ contributions

All the authors participated in the design of the study. SY and AR conceptualized the study, performed the statistical analysis and drafted the manuscript. TJ analyzed the results as well as draft and revise the manuscript. SY and SVG were involved in the development of all the components of the pipeline. RS reviewed the study, helped to draft the manuscript and gave final approval of the version to be published. All authors read and approved the final manuscript.

## Pre-publication history

The pre-publication history for this paper can be accessed here:

http://www.biomedcentral.com/1472-6947/14/13/prepub

## Supplementary Material

Additional file 1 Pipeline results for some of the drugs used in the study, as well as top 10 results for each of the drugs. Click here for file
