# Supplementary material for: A pipeline to extract drug-adverse event pairs from multiple data sources
Source: BMC Med Inform Decis Mak. 2014 Feb 24;14:13. doi: 10.1186/1472-6947-14-13 (PMC3936866; doi:10.1186/1472-6947-14-13)
Supplement: Additional file 1 — Pipeline results for some of the drugs used in the study, as well as top 10 results for each of the drugs. [file 1472-6947-14-13-S1.pdf]

Additional File 1

Pipeline results for some of the drugs used in the study, as well as top 10 results for each of the drugs

OLANZAPINE			
**Label**	**Blogs**	**AERS**	**PubMed (Literature)**
–	Anorexia nervosa	Anorexia nervosa	Anorexia nervosa
--	Depression	Depression; depression suicidal; depressive symptom	Depression; Depressive disorder; Depressive disorder, major; Depressive disorder, treatment resistant
--	Diabetes mellitus	Diabetes mellitus; diabetes mellitus, inadequate control;	Diabetes mellitus; diabetes mellitus, type 2
–	Eating disorders	Eating disorder	Eating disorders
–	Erectile dysfunction	–	--
Fever	Feeling cold	Feeling of body temperature change; Feeling drunk	--
--	Heart diseases	Heart rate decreased	--
--	Hyperglycemia	Diabetic hyperglycemic coma	Hyperglycemia; hyperglycemic hypersmolar nonketotic coma
–	Hypesthesia	Hypoaesthesia, oral	--
Weight gain; weight increased	Weight gain; Overweight	Overweight; weight increased	Weight gain; Overweight; weight increase
–	Paranoid disorders	Paranoia	Paranoid disorders
–	Sialohhrea	--	sialohhrea
–	Wounds and injuries	–	–

Table: Olanzapine: Comparative analysis of the label information from FDA and the positive scoring results of BCPNN on blogs, AERS and MEDLINE data. The blogs and literature sources support the AEs reported to AERS, which are not present in the drug label, such as eating disorders, diabetes mellitus, anorexia and depression. Unkown AEs (that do not show up in AERS), such as erectile dysfunction and heart diseases come up in blogs, while other AEs such as sialohhrea that come up in blogs are backed up by literature.

CIPROFLOXACIN			
**Label**	**Blogs**	**AERS**	**MEDLINE (Literature)**
–	Acne vulgaris	–	–
Confusion	Confusion	Confusional state	–
–	Dementia	–	–
–	Heartburn	–	–
Pain	Pain; Shoulder pain	Pain	–
Tendinitis	Tendinopathy	Tendonitis; tendon rupture; tendon pain; tendon injury; tendon disorder; tendon operation	tendinopathy

Table: Ciprofloxacin: Comparative analysis of the label information from FDA and the positive scoring results of BCPNN on blogs, AERS and MEDLINE data. Unkown AEs (that do not show up in AERS), such as Acne vulgaris, dementia and heartburn come up in blogs

CARBAMAZEPINE				
**Label**	**Blogs**	**AERS**	**PubMed (Literature)**	**SIDER**
–	Anxiety disorders	–	Anxiety disorders	Anxiety
–	Coma	Coma; Coma scale abnormal; Coma hepatic	Coma	Coma
Confusion	Confusion	Confusional state	Confusion	Confusion; Confusional state
–	Congenital abnormalities	Congenital cardiovascular anomaly; Congenital choroid plexus cyst; Congenital cystic kidney disease; Congenital diaphragmatic hernia; Congenital foot malformation; Congenital hand malformation; Congenital hip deformity; Congenital musculoskeletal anomaly; Congenital nail disorder; Congenital oral malformation; Congestive heart failure	–	Dermatitis ; Neurodermatitis ; Epidermal necrolysis ; Toxic epidermal necrolysis ; Exfoliative dermatitis
–	Contusions	–	–	–
–	Craniocerebral trauma	Craniotomy	Craniomandibular disorders	–
–	Cysts	Cystolytic hepatitis	Cyst removal	–
Exfoliative dermatitis	Exanthema	Acute generalised exanthematous pustulosis; Dermatitis; Dermatitis acneiform; Dermatitis allergic; Dermatitis bullous; Dermatitis contact; Dermatitis exfoliative; Dermatitis infected	Exanthema; Acute generalized exanthematous pustulosis; Allergic dermatitis; Dermatitis, exfoliative	Dermatitis ; Neurodermatitis ; Epidermal necrolysis ; Toxic epidermal necrolysis ; Exfoliative dermatitis
–	Hemorrhage	--	Basal ganglia hemorrhage; Cerebral hemorrhage, traumatic	–
–	Low back pain	–	–	Back pain
Lymphadenopathy;	Lymphoma	Lymphadenopathy; Lymphocyte morphology abnormal; Lymphocytic infiltration; Lymphopenia	Lymphatic diseases; Lymphocytoma cutis;	Lymphadenopathy ; Lymphoma
–	Memory disorders	Memory impairment	Memory disorders	Memory impairment
–	Neoplasms	Neoplasm; Neoplasm malignant; Oesophageal neoplasm; Brain neoplasm; Hepatic neoplasm; Hepatic neoplasm malignant; Malignant neoplasm progression	–	–
Osteoporosis	Osteoarthritis	Osteitis; Osteomalacia; Osteomyelitis; Osteonecrosis; Osteoporosis	Osteochondrodysplasias; Osteomalacia	Osteomalacia
Pancreatitis	Pancreatic cyst	Pancreatitis	–	Pancreatitis ; Pancreatic disorder
Personality change	Personality disorders	–	Personality disorders	Depersonalization
–	Seizures	–	Seizures; Seizures, febrile	Convulsive seizures
–	Weight; weight loss	–	Weight gain	Weight gain ; Weight increased

Table: Carbamazepine: Comparative analysis of the label information from FDA and the positive scoring results of BCPNN on blogs, AERS and PubMed data

Tables: The following tables contain the Top 10 scoring adverse events for each of the drugs used in this study. The scores here are the variance values obtained from running the BCPNN algorithm on the drug-AE pairs for each source.

ASPIRIN					
AERS		Blogs		TPX	
Event	BCPNN IC Variance	Event	BCPNN IC Variance	Event	BCPNN IC Variance
FLUSHING	3.0453446021	HEMORRHAGE	2.2974781367	ANTIPLATELET THERAPY	6.4498501796
COLITIS COLLAGENOUS	2.967777029	STROKE	1.9440919537	ASTHMA, ASPIRIN-INDUCED	6.2465967694
THERAPEUTIC AGENT TOXICITY	2.8940435493	ASTHMA	1.7849414476	PLATELET AGGREGATION	4.9990559736
GASTROINTESTINAL ULCER	2.8231226105	CONTUSIONS	1.7056384898	BLEEDING TIME	4.8647435024
THROMBOSIS IN DEVICE	2.6858252696	ULCER	1.6889750542	REYE SYNDROME	4.7192352524
FEELING HOT	2.6826249441	STOMACH ULCER	1.4228170456	ACETAMINOPHEN	4.6032193367
GASTRIC ULCER HAEMORRHAGE	2.5873478486	HBP	1.4176260231	ACUTE CORONARY SYNDROME	4.5350807860
SKIN BURNING SENSATION	2.5589130946	COLD	1.3658990352	ANGINA, UNSTABLE	4.5321633114
ANGIOEDEMA	2.4875946967	FLUSHING	1.3159626717	WIDAL SYNDROME	4.5238714090
GASTRIC ULCER	2.4858617938	ARTHRITIS	1.3041053189	ERYTHROMELALGIA	4.4253923685

Table: Top 10 for Aspirin

BUPROPION					
AERS		Blogs		TPX	
Event	BCPNN IC Variance	Event	BCPNN IC Variance	Event	BCPNN IC Variance
PSORIASIS	3.0858333183	SMOKING	2.8487222833	TOBACCO USE DISORDER	6.7616174378
TINNITUS	3.0003865794	INTERACTION	2.0278154524	SUBSTANCE WITHDRAWAL SYNDROME	5.6160138431
CRYING	2.9716117286	WEIGHT LOSS	1.9125982688	DEPRESSIVE DISORDER, MAJOR	5.0765060025
DEPRESSION	2.9671908886	TREMOR	1.7558640559	SMOKING	4.6561130303
DRUG INEFFECTIVE	2.9603666179	HYPOTHYROIDISM	1.6536311321	HAM	4.4458534794
THERAPEUTIC RESPONSE DECREASED	2.7367299785	CHRONIC PAIN	1.6142569642	ATTENTION DEFICIT DISORDER WITH HYPERACTIVITY	4.2252387708
ADVERSE DRUG REACTION	2.695957113	DEPRESSION	1.4633641249	SLEEP INITIATION AND MAINTENANCE DISORDERS	4.2093356907
GRAND MAL CONVULSION	2.6583368082	FIBROMYALGIA	1.4626978699	SEXUAL DYSFUNCTION	4.1191726291
THERAPEUTIC RESPONSE UNEXPECTED WITH DRUG SUBSTITUTION	2.5137356129	AIRWAY OBSTRUCTION	1.4014082071	OVERDOSE	4.0895995908
CONVULSION	2.4652329995	SHAKING	1.4014082071	DEPRESSIVE DISORDER, TREATMENT-RESISTANT	4.0677836593

Table: Top 10 for Bupropion

CARBAMAZEPINE					
AERS		Blogs		TPX	
Event	BCPNN IC Variance	Event	BCPNN IC Variance	Event	BCPNN IC Variance
ANTICONVULSANT DRUG LEVEL INCREASED	3.8394557589	SEIZURES	2.1831882505	TRIGEMINAL NEURALGIA	5.7366534252
DRUG RASH WITH EOSINOPHILIA AND SYSTEMIC SYMPTOMS	3.5911285749	EXANTHEMA	1.8627395675	EPILEPSIES, PARTIAL	5.5948458460
ANTICONVULSANT DRUG LEVEL ABOVE THERAPEUTIC	3.2657628102	LYMPHOMA	1.4381580775	STEVENS-JOHNSON SYNDROME	5.3815350109
ANTICONVULSANT DRUG LEVEL DECREASED	3.1926598078	LOW BACK PAIN	1.3923651696	ISAACS SYNDROME	5.3032705984
ABORTION INDUCED	3.1373775719	CONGENITAL ABNORMALITIES	1.3923651696	GLOSSOPHARYNGEAL NERVE DISEASES	5.1246367023
ANTICONVULSANT DRUG LEVEL BELOW THERAPEUTIC	3.0830808142	NEOPLASMS	1.3812234183	EPIDERMAL NECROLYSIS, TOXIC	5.0291405655
CAESAREAN SECTION	3.0482773803	ANXIETY DISORDERS	1.1070229426	EPILEPSY	4.9773218189
EPILEPSY	3.0298491782	PRURITUS	1.1070229426	EPILEPSY, ABSENCE	4.9438297118
CONVULSION	2.9167016489	OSTEOARTHRITIS	1.070503923	EPILEPSY, GENERALIZED	4.9300947149
ALANINE AMINOTRANSFERASE INCREASED	2.9049714934	PANCREATIC CYST	0.9004897912	BIPOLAR DISORDER	4.8501453144

Table: Top 10 for Carbamazepine

CIPROFLOXACIN					
AERS		Blogs		TPX	
Event	BCPNN IC Variance	Event	BCPNN IC Variance	Event	BCPNN IC Variance
TENDON RUPTURE	4.1146259253	UTI	2.8856797854	MBC	4.6062736746
TENDONITIS	4.0060829925	DIVERTICULITIS	2.2799989819	CROSS RESISTANCE	4.4062217226
TENDON PAIN	3.6892637795	ACNE VULGARIS	1.7510888515	DYSENTERY, BACILLARY	4.3850295544
MULTIPLE-DRUG RESISTANCE	3.5299714532	ARTHRALGIA	1.6148411075	OTITIS EXTERNA	4.3473788710
ARTHRALGIA	3.492025139	SMOKING	1.612155782	COMMUNITY-ACQUIRED INFECTIONS	4.3167751507
TENDON DISORDER	3.4487011137	VAGINITIS	1.4865210867	URINARY TRACT INFECTIONS	4.2543152131
ROTATOR CUFF SYNDROME	3.2285729273	DEMENTIA	1.4865210867	MRSA	4.1979997347
CLOSTRIDIUM DIFFICILE COLITIS	3.151939512	COLITIS	1.4865210867	MINIMUM INHIBITORY CONCENTRATION	4.1863790454
COLECTOMY	3.1377574934	TENDINOPATHY	1.4865210867	SOFT TISSUE INFECTIONS	4.1863232530
NEUROPATHY PERIPHERAL	3.0132904631	BITES AND STINGS	1.3943698407	TYPHOID FEVER	4.1574971886

Table: Top 10 for Ciprofloxacin

IBUPROFEN					
AERS		Blogs		TPX	
Event	BCPNN IC Variance	Event	BCPNN IC Variance	Event	BCPNN IC Variance
CHOLECYSTITIS CHRONIC	3.2168422753	ARTHRITIS	2.8160182569	ACETAMINOPHEN	5.2496866778
KOUNIS SYNDROME	2.9815043448	RA	2.4885591332	DUCTUS ARTERIOSUS, PATENT	4.8635855143
TOXIC EPIDERMAL NECROLYSIS	2.8890346654	PAIN	2.3836377566	ACUTE PAIN	4.4671583670
OSTEOARTHRITIS	2.8760959875	HEMORRHAGE	2.1607207909	DYSMENORRHEA	4.3249604300
MENINGITIS ASEPTIC	2.7728412562	OSTEOARTHRITIS	1.9773610941	PAIN, POSTOPERATIVE	3.9313820449
NECROTISING COLITIS	2.7516860221	HEADACHE	1.777748493	MENINGITIS, ASEPTIC	3.8782863105
ACCIDENTAL DRUG INTAKE BY CHILD	2.7513002641	INFLAMMATION	1.7614007628	OSTEOARTHRITIS	3.5868790708
RENAL TUBULAR ACIDOSIS	2.7461620025	STOMACH ULCER	1.6554329992	ANALGESIA	3.2560627200
GALLBLADDER DISORDER	2.7344194421	HOT FLASHES	1.6301140771	ENTEROCOLITIS, NECROTIZING	3.1247380905
HEPATITIS CHOLESTATIC	2.7051913567	ARTHRALGIA	1.6301140771	DYSMENORRHOEA	3.0808637411

Table: Top 10 for Ibuprofen

MORPHINE					
AERS		Blogs		TPX	
Event	BCPNN IC Variance	Event	BCPNN IC Variance	Event	BCPNN IC Variance
DRUG ABUSE	4.2642551879	PAIN	1.5965513429	MORPHINE DEPENDENCE	7.1356766869
ACUTE CHEST SYNDROME	3.9896519753	PAIN MANAGEMENT	0.9969507103	POSTOPERATIVE ANALGESIA	5.9041759822
DRUG DIVERSION	3.7848502635	HASHIMOTO DISEASE	0.9969507103	OPIATES	5.8450420720
SUBSTANCE ABUSE	3.7711101144	BREATHLESSNESS	0.9969507103	OPIOID-RELATED DISORDERS	5.4082024508
ACCIDENTAL DEATH	3.5380803319	VIOLENT	0.995428479	BREAKTHROUGH PAIN	5.2295675299
CARDIO-RESPIRATORY ARREST	3.2009993755	AIRWAY OBSTRUCTION	0.995428479	PAIN, POSTOPERATIVE	5.1450496056
BREAKTHROUGH PAIN	3.173061935	STRESS	0.9939078521	ANALGESIA	4.9912679855
PHARMACEUTICAL PRODUCT COMPLAINT	2.9664441881	FIBROMYALGIA	0.9727856709	HYPERALGESIA	4.6437710157
RESPIRATORY DEPRESSION	2.9511565422	ULCER	0.968299444	SUBSTANCE WITHDRAWAL SYNDROME	4.5433001387
ACCIDENTAL OVERDOSE	2.9169495653	ANXIETY	0.968299444	VISCERAL PAIN	4.3657303159

Table: Top 10 for Morphine

OLANZAPINE					
AERS		Blogs		TPX	
Event	BCPNN IC Variance	Event	BCPNN IC Variance	Event	BCPNN IC Variance
HOSPITALISATION	3.6824877733	DIABETES MELLITUS	1.96163018	SCHIZOPHRENIA	5.7849079521
LEUKOPENIA	3.2803137933	OVERWEIGHT	1.8868082131	BIPOLAR DISORDER	5.7118297960
METABOLIC SYNDROME	3.1617168832	FEELING COLD	1.4527063824	PSYCHOMOTOR AGITATION	5.6353982109
NEUTROPENIA	3.1562789595	HYPERGLYCEMIA	1.4527063824	PSYCHOTIC DISORDERS	5.4453125779
DIABETES MELLITUS	3.0309265138	ANOREXIA NERVOSA	1.4527063824	NEUROLEPTIC MALIGNANT SYNDROME	5.3997509746
OFF LABEL USE	3.0039352761	ERECTILE DYSFUNCTION	1.4111846544	WEIGHT GAIN	5.3027752842
SCHIZOPHRENIA	2.8965197591	PERSONALITY DISORDERS	1.3708245938	MOVEMENT DISORDERS	4.8641047999
WHITE BLOOD CELL COUNT DECREASED	2.8842888897	WEIGHT GAIN	1.2974368552	DEPRESSIVE DISORDER, TREATMENT-RESISTANT	4.7405104427
GRANULOCYTOPENIA	2.6981219136	EATING DISORDERS	1.2933415596	SCHIZOPHRENIA, PARANOID	4.6771690090
NEUTROPHIL COUNT DECREASED	2.667754068	WOUNDS AND INJURIES	0.9286602503	HYPERPROLACTINEMIA	4.5234303792

Table: Top 10 for Olanzapine

PAROXETINE					
AERS		Blogs		TPX	
Event	BCPNN IC Variance	Event	BCPNN IC Variance	Event	BCPNN IC Variance
CONGENITAL ANOMALY	3.1300167523	ANXIETY DISORDERS	1.5594343083	HAM	5.6353454077
ATRIAL SEPTAL DEFECT	3.1224780702	PHOBIC DISORDERS	1.5388697964	DEPRESSIVE DISORDER, MAJOR	5.5424233484
CARDIAC MURMUR	2.9842039722	HOT FLASHES	1.4317963274	PANIC DISORDER	5.3198511794
BICUSPID AORTIC VALVE	2.9562774445	ANXIETY	1.2333725457	SEROTONIN SYNDROME	5.2905075543
CRANIOSYNOSTOSIS	2.9448015338	BREAST NEOPLASMS	1.2260431853	OBSESSIVE-COMPULSIVE DISORDER	5.2394610320
AORTA HYPOPLASIA	2.9415590337	EMBOLISM	1.2260431853	ANXIETY DISORDERS	4.9782065619
ANOMALOUS PULMONARY VENOUS CONNECTION	2.9311732694	DEPRESSION	1.2142281183	DEPRESSION	4.6737059187
AORTIC VALVE INCOMPETENCE	2.8684022651	SHOCK	1.114507919	DEPRESSIVE DISORDER, TREATMENT-RESISTANT	4.3473087277
ARNOLD-CHIARI MALFORMATION	2.8513430628	GAD	1.114507919	HOT FLASHES	4.1215071386
ACTIVATION SYNDROME	2.8316456249	SUBSTANCE WITHDRAWAL SYNDROME	1.108942083	DEPRESSIVE DISORDER	4.0962192642

Table: Top 10 for Paroxetine

ROSIGLITAZONE					
AERS		Blogs		TPX	
Event	BCPNN IC Variance	Event	BCPNN IC Variance	Event	BCPNN IC Variance
HEART INJURY	1.5296202559	HEART DISEASES	1.9460752101	DIABETES MELLITUS, TYPE 2	5.5082168188
CARDIOVASCULAR DISORDER	1.5209213985	DIABETES MELLITUS	1.6536311321	INSULIN RESISTANCE	5.4173083948
MYOCARDIAL ISCHAEMIA	1.5186640529	CORONARY ARTERY DISEASE	1.564503778	POLYCYSTIC OVARY SYNDROME	4.1594095171
CORONARY ARTERY DISEASE	1.5140236985	NAUSEA	1.1597541931	DYSLIPIDEMIAS	4.1067290058
CARDIAC FAILURE CONGESTIVE	1.4984233756	WEIGHT GAIN	0.0444301566	LIPODYSTROPHY	3.9718676602
MYOCARDIAL INFARCTION	1.4908778821	LYME DISEASE	-0.0136713334	HYPERINSULINISM	3.8471096081
CORONARY ARTERY BYPASS	1.4609991226	CLOTTING	-0.0136713334	METABOLIC SYNDROME X	3.7648511713
ARTERIOSCLEROSIS	1.4521357761	PAIN MANAGEMENT	-0.0136713334	HYPERGLYCEMIA	3.5928127873
ACUTE CORONARY SYNDROME	1.4461843057	MACULAR DEGENERATION	-0.0136713334	WEIGHT GAIN	3.5540271397
ISCHAEMIC CARDIOMYOPATHY	1.439707607	CONSTRICTION, PATHOLOGIC	-0.0136713334	FATTY LIVER	3.3768183250

Table: Top 10 for Rosiglitazone

TRAZODONE					
AERS		Blogs		TPX	
Event	BCPNN IC Variance	Event	BCPNN IC Variance	Event	BCPNN IC Variance
CARDIAC ARREST	2.751062765	BACK PAIN	2.0113279316	SLEEP INITIATION AND MAINTENANCE DISORDERS	5.5423900660
COMPLETED SUICIDE	2.4845999228	SLEEP INITIATION AND MAINTENANCE DISORDERS	1.4252916669	PRIAPISM	5.2601858298
SUICIDAL IDEATION	2.3695413431	CONDYLOMATA ACUMINATA	1.3983875588	OVERDOSE	4.5116173724
PANCREATITIS	2.3008694939	BACK INJURIES	1.3983875588	DEPRESSIVE DISORDER	4.3337886487
NAUSEA	2.1046610232	BONE DISEASES	1.3983875588	SEROTONIN SYNDROME	4.2508757568
ABORTION SPONTANEOUS	1.9980906149	BP	1.3235030585	DEPRESSIVE DISORDER, MAJOR	3.7483223255
SEROTONIN SYNDROME	1.8328170063	NARCOLEPSY	1.2861894858	HYPOTENSION, ORTHOSTATIC	3.6172144589
INTENTIONAL OVERDOSE	1.8326981157	AGORAPHOBIA	1.2861894858	DEPRESSION	3.5945815197
PAIN	1.7794386401	SLEEP DEPRIVATION	1.2332014951	ERECTILE DYSFUNCTION	3.4512055897
TYPE 2 DIABETES MELLITUS	1.7710158907	OCD	1.2261921126	OBSESSIVE-COMPULSIVE DISORDER	3.4396403539

Table: Top 10 for Trazodone

WARFARIN					
AERS		Blogs		TPX	
Event	BCPNN IC Variance	Event	BCPNN IC Variance	Event	BCPNN IC Variance
INTERNATIONAL NORMALISED RATIO DECREASED	3.1727941794	INR	4.2516503259	VITAMIN K	6.1945919883
INTERNATIONAL NORMALISED RATIO FLUCTUATION	3.1642485602	HEMORRHAGE	2.8983756918	BCR	5.8376604098
INTERNATIONAL NORMALISED RATIO ABNORMAL	3.1524911849	STROKE	2.5276002364	THROMBOEMBOLISM	5.7963964427
INTERNATIONAL NORMALISED RATIO INCREASED	3.1363362971	PULMONARY EMBOLISM	2.3963773011	HYPOPROTHROMBINEMIAS	5.6662604708
CALCIPHYLAXIS	2.8692951662	NEOPLASMS	2.1529503539	VENOUS THROMBOEMBOLISM	5.6468512140
BLUE TOE SYNDROME	2.8692951662	ATRIAL FIBRILLATION	2.1333428953	ATRIAL FIBRILLATION	5.4179635146
FOOD INTERACTION	2.8474983862	PROTEIN S DEFICIENCY	1.87157852	VITAMIN K DEFICIENCY	5.2157603210
SKIN NECROSIS	2.7952072615	ANTIPHOSPHOLIPID SYNDROME	1.87157852	ANTICOAGULANT THERAPY	5.2066920622
PREGNANCY	2.7364463059	MUSCLE CRAMP	1.6446553998	PROTHROMBIN	5.1233465107
HAEMORRHAGE INTRACRANIAL	2.7025126897	SMOKING	1.6184685316	PROTEIN C DEFICIENCY	4.9709289811

Table: Top 10 for Warfarin

ZIPRASIDONE					
AERS		Blogs		TPX	
Event	BCPNN IC Variance	Event	BCPNN IC Variance	Event	BCPNN IC Variance
TARDIVE DYSKINESIA	4.2677078217	VOMITING	1.9460228686	SCHIZOPHRENIA	5.7401521972
DYSTONIA	4.0558212672	TREMOR	1.831079029	QT INTERVAL	5.6217692996
EXTRAPYRAMIDAL DISORDER	3.7617945087	PSYCHOTIC DISORDERS	1.6030569698	BIPOLAR DISORDER	5.6013438249
AKATHISIA	3.6922456683	ANXIETY	1.5339814308	PSYCHOTIC DISORDERS	5.4158359358
BRUXISM	3.3493728888	SNEEZING	1.3794022687	MOVEMENT DISORDERS	5.1004160139
DYSKINESIA	3.2568276057	ERECTILE DYSFUNCTION	1.3169129592	WEIGHT GAIN	5.0058044895
GALACTORRHOEA	3.034870682	SCHIZOPHRENIA	1.3169129592	PSYCHOMOTOR AGITATION	4.9158544547
ELECTROCARDIOGRAM QT PROLONGED	3.0348478874	STRESS DISORDERS, POST-TRAUMATIC	1.2744503122	HYPERPROLACTINEMIA	4.5196414562
SEDATION	2.9891069927	MENTAL DISORDERS	1.1995114761	TORSADES DE POINTES	4.1031400764
BIPOLAR DISORDER	2.956694167	TASTE DISORDERS	1.1995114761	DYSTONIA	4.0131882497

Table: Top 10 for Ziprasidone
